# The emerging discipline has emerged: cardio-oncology as a medical specialty

**DOI:** 10.3389/fcvm.2026.1954059

**Published:** 2026-10-28

**Authors:** Yohannes T. Ghebre, Derek R. Shyr, Pius S. Fasinu

**Affiliations:** 1 Marnix E. Heersink School of Medicine, The University of Alabama at Birmingham, Birmingham, AL, United States; 2 Department of Radiation Oncology, The University of Texas Health Science Center at San Antonio, San Antonio, TX, United States; 3 Mays Cancer Center, The University of Texas Health Science Center at San Antonio, San Antonio, TX, United States; 4 Department of Medical Education, The University of Alabama at Birmingham Heersink School of Medicine, Birmingham, AL, United States

**Keywords:** cancer therapy-related cardiovascular toxicity, cardio-oncology, cardiovascular medicine, multidisciplinary care, oncology, precision medicine, survivorship

## Abstract

Remarkable advances in cancer therapeutics have transformed many malignancies into chronic, manageable diseases, creating an unprecedented population of long-term cancer survivors. However, these gains have been accompanied by a growing burden of cardiovascular disease resulting from both cancer therapies and shared biological mechanisms between cancer and cardiovascular disease. Cardio-oncology has emerged to address this challenge by integrating cardiovascular prevention, surveillance, diagnosis, and treatment throughout the range of cancer care. What began as a consultative niche focused largely on anthracycline-induced cardiomyopathy has rapidly evolved into a multidisciplinary specialty supported by international guidelines, dedicated clinical programs, structured surveillance strategies, specialized training initiatives, and expanding research networks. This perspective argues that cardio-oncology has crossed the threshold from an emerging field to an established medical discipline. We discuss the milestones that define its maturation, the challenges that remain, and the priorities required to ensure equitable implementation worldwide.

## Introduction

A patient diagnosed with Human Epidermal growth factor Receptor 2 (HER2)-positive breast cancer today faces a vastly different prognosis than a similar patient two decades ago. Modern targeted therapies have dramatically improved survival (1–3), yet they introduce new cardiovascular risks that may compromise treatment (4–6), reduce quality of life, or contribute to long-term mortality. The challenge is no longer simply curing cancer—it is preserving cardiovascular health while achieving optimal oncologic outcomes (5).

This evolving reality has fundamentally reshaped the relationship between oncology and cardiovascular medicine. Cardio-oncology has emerged in response to the recognition that cancer and cardiovascular disease are increasingly interconnected through shared risk factors, common biological pathways, and treatment-related toxicities (4–8). The field now extends well beyond the management of chemotherapy-induced heart failure, encompassing prevention, risk stratification, surveillance, acute cardiovascular complications, survivorship, and long-term cardiovascular care (9–14) (Figure 1).

**Figure 1:**
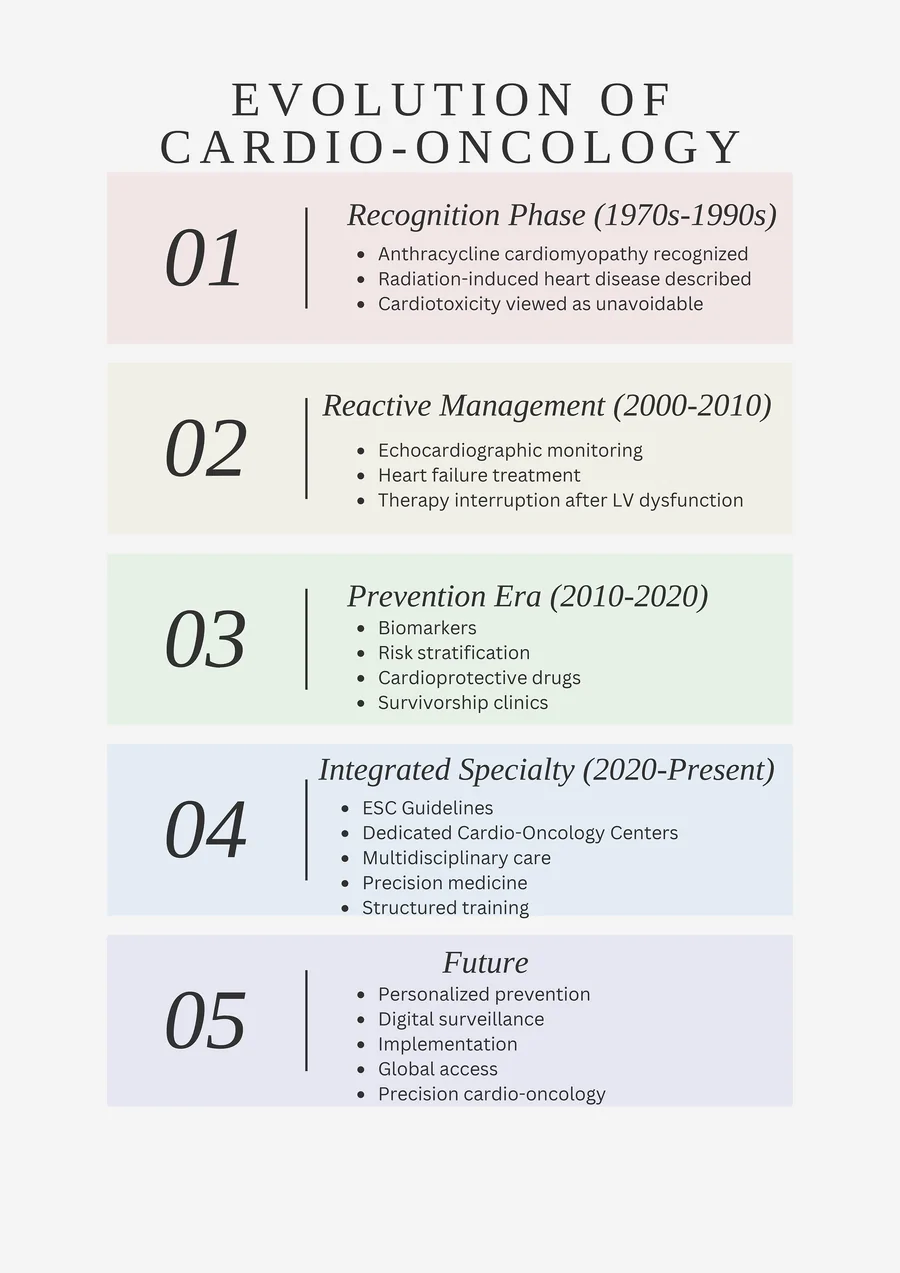
Evolution of Cardio-Oncology: From cardiotoxicity to comprehensive cardiovascular care. The timeline shows how the field has progressed from recognizing anthracycline-induced heart failure in the 1970s–1990s to incorporating cardiovascular risk assessment, surveillance, prevention, survivorship, precision medicine, and multidisciplinary care throughout the cancer field.

The question is no longer whether cardio-oncology is an emerging discipline. Rather, the evidence suggests that it has already emerged as a mature specialty with its own clinical framework, scientific agenda, professional identity, and educational infrastructure. In this perspective, we use the term “mature specialty” to describe a field that has progressed beyond recognition of a clinical problem to possess several defining features of an established discipline: standardized clinical guidance, dedicated multidisciplinary services, an identifiable professional community, specialty-specific education and training, organized research networks, and mechanisms for quality assessment and professional recognition. Cardio-oncology increasingly meets these criteria, although maturation remains incomplete in areas such as standardized certification, workforce development, and equitable global access.

## Why cardio-oncology became necessary

The success of modern oncology has unintentionally created a new population of patients living long enough to experience cardiovascular complications of cancer therapy (1–3). Improvements in screening, precision therapeutics, immunotherapy, and supportive care have substantially increased cancer survival, making cardiovascular disease one of the leading causes of morbidity and mortality among cancer survivors (2, 3, 15).

At the same time, cancer and cardiovascular disease share many traditional risk factors, including aging, hypertension, diabetes, obesity, tobacco exposure, chronic inflammation, and metabolic dysfunction (4, 6, 14). Beyond these common risk factors, emerging evidence demonstrates biological crosstalk between malignancy and cardiovascular disease, suggesting that the two conditions frequently coexist through overlapping mechanisms rather than sheer coincidence.

The result is a growing population of increasingly complex patients requiring expertise that neither oncology nor cardiology independently provides.

## The defining moment: international guidelines

Perhaps the uber marker of specialty maturation was the publication of the 2022 European Society of Cardiology (ESC) Cardio-Oncology Guidelines, the first comprehensive international clinical practice guideline dedicated entirely to cardio-oncology (5).

These guidelines fundamentally shifted the field from reactive management toward proactive cardiovascular care throughout the cancer field. They standardized terminology, introduced structured cardiovascular risk assessment before treatment initiation, established surveillance recommendations based on therapy-specific risk, and emphasized multidisciplinary decision-making aimed at minimizing unnecessary interruptions of potentially life-saving cancer therapy (5).

Equally important, the guidelines recognized cardiovascular toxicity as a dynamic process rather than an isolated complication, promoting the concept of continuous cardiovascular care before, during, and after cancer treatment (Figure 2).

**Figure 2:**
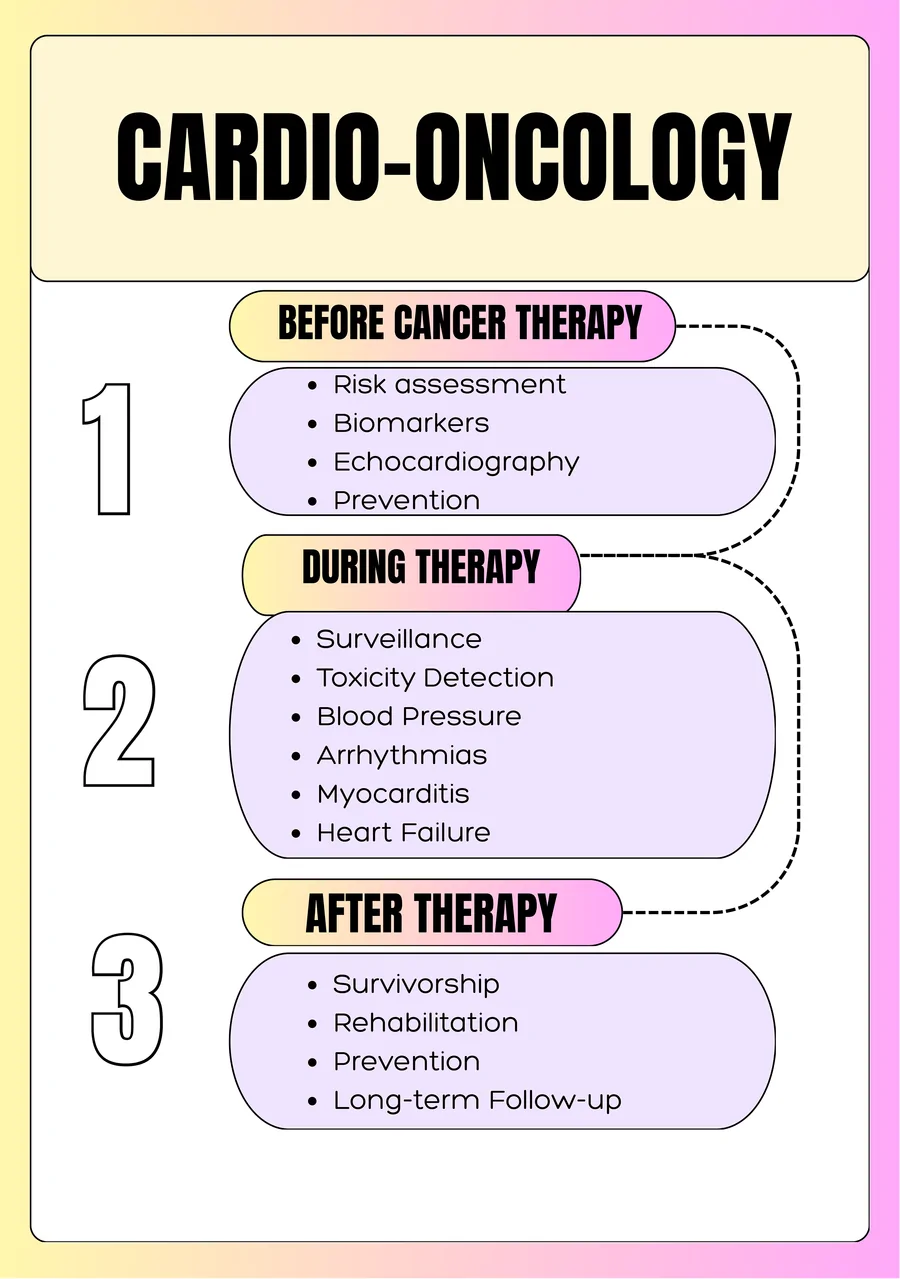
The contemporary scope of Cardio-Oncology. The figure reinforces the central message that cardio-oncology is no longer simply about “cardiotoxicity,” but rather provides comprehensive cardiovascular care across the entire cancer journey.

Since their publication, additional consensus statements from international organizations including the International Cardio-Oncology Society (IC-OS) have further reinforced standardized clinical pathways, demonstrating increasing global alignment around cardio-oncology practice (8–10).

## From cardiotoxicity to cardiovascular prevention

Early cardio-oncology focused predominantly on managing anthracycline-induced heart failure after irreversible myocardial injury had already occurred (16). Today's philosophy is fundamentally different. Modern cardio-oncology emphasizes prevention.

Baseline cardiovascular risk assessment now guides individualized surveillance strategies incorporating echocardiography, cardiac biomarkers, and advanced imaging. High-risk patients may receive cardioprotective pharmacotherapy before measurable cardiac dysfunction develops, while surveillance schedules are increasingly tailored according to cancer type, therapeutic regimen, and individual cardiovascular risk (Table 1).

**Table 1:** Milestones in the evolution of Cardio-Oncology. Timeline highlighting major milestones surrounding the genesis and progression of Cardio-Oncology.

Year	Milestone	Significance
1970s	Anthracycline cardiomyopathy recognized	First appreciation that cancer therapy can produce permanent cardiac injury
1980s–1990s	Radiation-induced cardiovascular disease characterized	Expanded understanding beyond chemotherapy-associated toxicity
Early 2000s	HER2-targeted therapy-associated cardiotoxicity identified	Highlighted reversible forms of cardiac dysfunction and need for surveillance
2009	Seminal paper in JACC (Yeh & Bickford)	Established cardio-oncology as an emerging interdisciplinary field
2010s	Dedicated cardio-oncology clinics established	Transition from individual consultations to organized clinical programs
2014	International Cardio-Oncology Society (IC-OS) founded	Created an international professional organization dedicated to the specialty
2015	JACC Council Clinical Perspective	Highlighted the need for specialized cardio-oncology services, education, training, and professional organization
2017–2021	Expansion of cardio-oncology fellowship training and competency statements	Formalized education and workforce development
2019	JACC: CardioOncology launched	Established a dedicated peer-reviewed scientific journal
2022	ESC Cardio-Oncology Guidelines published	Standardized clinical practice across the cancer field and marked specialty maturation
2023–2026	Growth of international registries, AI-assisted surveillance, and Center of Excellence programs	Represents the transition from an emerging field to an established global specialty

This preventive paradigm mirrors successful strategies in preventive cardiology and reflects a broader transition toward precision medicine in oncology (5, 17).

Rather than asking whether cardiac dysfunction has developed, clinicians increasingly ask whether it can be anticipated and prevented.

## The expanding clinical scope

The modern cardio-oncology practice encompasses a remarkably diverse spectrum of cardiovascular complications.

Anthracyclines and HER2-directed therapies remain important causes of cancer therapy-related left ventricular dysfunction (11, 12), but they now represent only part of the specialty's clinical landscape. Meanwhile, immune checkpoint inhibitors have introduced immune-mediated myocarditis (18–20), one of the most feared complications in modern oncology because of its high mortality despite relative rarity. By contrast, targeted therapies such as vascular endothelial growth factor (VEGF) inhibitors (21) and tyrosine kinase inhibitors (TKIs) (22) have expanded the field into hypertension management, vascular dysfunction, thromboembolism, and accelerated atherosclerosis.

Moreover, radiation-associated cardiovascular disease (23) remains an important long-term concern among cancer survivors, often manifesting years or decades after treatment. Meanwhile, atrial fibrillation, heart failure, thromboembolism, pulmonary hypertension, and ischemic heart disease (24) increasingly require coordinated management within oncology populations.

Collectively, these conditions illustrate how cardio-oncology has evolved into comprehensive cardiovascular medicine practiced in the context of cancer rather than simply the management of chemotherapy toxicity. Importantly, the evidence base is not uniform across this spectrum: anthracycline-, HER2-, VEGF inhibitor-, and radiation-associated toxicities are relatively well characterized, whereas optimal screening and management for newer or less common conditions, including immune checkpoint inhibitor myocarditis, continue to evolve.

## Cardio-oncology as a healthcare system

Dedicated cardio-oncology clinics have expanded rapidly across academic medical centers worldwide (25–27). Multidisciplinary teams may include cardiologists, oncologists, hematologists, pharmacists, advanced practice providers, imaging specialists, and nursing professionals working within integrated care pathways.

Professional organizations have established international registries, collaborative research networks, educational programs, and center recognition initiatives that promote quality improvement and standardization.

Tumor boards increasingly incorporate cardiovascular expertise, survivorship programs routinely include cardiovascular risk assessment, and institutional protocols now integrate cardiovascular surveillance into routine oncology care. These developments represent institutionalization—a defining characteristic of a mature medical specialty (8, 25). However, the scale and composition of dedicated services vary substantially across institutions, and comparative evidence linking dedicated cardio-oncology programs to improved clinical outcomes remains limited.

## Building a professional identity

Another hallmark of specialty maturation is the emergence of dedicated educational pathways.

Cardio-oncology requires competencies extending beyond traditional cardiovascular medicine, including familiarity with rapidly evolving oncology therapeutics, surveillance imaging, biomarker interpretation, clinical trial design, survivorship care, and multidisciplinary communication.

Recognizing this need, professional societies have begun developing structured curricula, fellowship experiences, competency frameworks, and continuing medical education (CME) programs specifically devoted to cardio-oncology (9, 10, 28). Although standardized certification pathways remain limited, the trajectory resembles the historical evolution of disciplines such as interventional cardiology, electrophysiology, and critical care medicine. The specialty is increasingly defining not only what practitioners should know but how future generations should be trained. This professional identity is also reflected in the establishment of the International Cardio-Oncology Society, early recognition of the field in major cardiovascular publications, including the 2015 JACC Council Clinical Perspective outlining a roadmap for cardio-oncology development (10), and the American College of Cardiology's dedicated Cardio-Oncology Member Section (33). Specialty-specific certification has also emerged through IC-OS individual certification and Center of Excellence designation, although universally standardized certification remains limited (34). The evolution of cardio-oncology as a distinct medical specialty also raises important questions regarding formal professional certification and accreditation. Alvarez-Cardona and colleagues (9) highlighted the need to consider multiple pathways for establishing standardized competencies in cardio-oncology, including certification mechanisms facilitated by professional organizations such as the American College of Cardiology (ACC) and the IC-OS. Notably, IC-OS has already implemented individual certification examinations and institutional recognition through its Centers of Excellence program (34). However, these initiatives remain distinct from certification administered through the American Board of Medical Specialties (ABMS) and its Member Boards. Within the ABMS framework, the American Board of Internal Medicine (ABIM) currently offers certification in cardiovascular disease and medical oncology, as well as cardiovascular subspecialties including adult congenital heart disease, advanced heart failure and transplant cardiology, and clinical cardiac electrophysiology. Cardio-oncology, however, is not currently recognized as a distinct ABIM subspecialty certification (35, 36). This distinction underscores the difference between the growing professional and institutional recognition of cardio-oncology and its formal recognition within the established U.S. medical specialty-certification system.

## Remaining challenges

Despite remarkable progress, important gaps remain.

Most recommendations continue to rely heavily on observational studies and expert consensus rather than randomized clinical trials (5, 29). Questions regarding optimal initiation of cardioprotective therapy, duration of surveillance, management of asymptomatic biomarker abnormalities, and long-term cardiovascular outcomes remain incompletely answered. Furthermore, artificial intelligence, multimodal imaging, circulating biomarkers, genomics, and proteomics offer considerable promise for improving individualized risk prediction, but their clinical integration remains in its infancy. Addressing these gaps will require prospective multicenter studies, pragmatic trials, and international registries that evaluate cardiovascular outcomes alongside cancer-treatment modalities and patient-reported outcomes.

Equally important are disparities in access. Dedicated cardio-oncology programs remain concentrated in major academic centers, while many patients worldwide continue to receive cancer treatment without structured cardiovascular assessment. In this regard, workforce development and equitable access are important factors that need careful implementation. Finally, survivorship care must evolve beyond monitoring for toxicity toward lifelong cardiovascular health promotion, integrating prevention, rehabilitation, lifestyle modification, and chronic disease management (30–32). Scalable approaches may include tele-cardio-oncology, standardized referral pathways, and shared-care models with general cardiology, primary care, oncology, and survivorship services.

## The next frontier: operationalizing cardio-oncology as a fully integrated specialty

As cardio-oncology transitions from an emerging discipline to an established specialty, the next challenge is not validation but implementation. Broader formal recognition within health systems, academic institutions, and professional organizations should be accompanied by standardized curricula, fellowship pathways, clearly defined competencies, and competency-based certification that prepare the future workforce. Equally important is the adoption of an integrated care model in which cardiovascular expertise is embedded across the entire cancer field—from cancer diagnosis and baseline cardiovascular risk assessment, through precision surveillance during treatment, to early intervention, survivorship, and lifelong cardiovascular care. Rather than functioning primarily as a referral service for treatment-related cardiotoxicity, future cardio-oncology programs should become an integral component of routine cancer management, leveraging multidisciplinary care, digital health, biomarkers, advanced imaging, artificial intelligence, and precision medicine to deliver proactive, personalized cardiovascular care for every patient with cancer. Achieving this vision will require coordinated efforts from professional societies, accrediting authorities, healthcare systems, and policymakers to establish consistent standards while ensuring equitable access across diverse practice settings. An important future milestone in the continued maturation of cardio-oncology may be its formal recognition within the ABMS certification framework. The ABMS Committee on Certification (COCERT) provides an established mechanism for reviewing and evaluating proposals submitted by ABMS Member Boards for new specialty and subspecialty certificates and focused practice designations (37). Accordingly, a potential pathway toward formal recognition of cardio-oncology would involve engagement with the appropriate ABMS Member Board or Boards, such as the American Board of Internal Medicine, to develop and submit a proposal through the established certification-review process. Importantly, COCERT does not independently establish new subspecialties, and any proposal would require appropriate Member Board sponsorship and approval through ABMS governance procedures. If pursued and ultimately approved, such recognition could provide a framework for standardized physician training requirements, competency assessment, certification examinations, and continuing professional development. Nevertheless, formal ABMS recognition should be viewed as a potential future milestone rather than a prerequisite for the continued clinical development and integration of cardio-oncology. Progress toward this goal should occur alongside efforts to expand workforce capacity, strengthen multidisciplinary care, and improve equitable access to specialized cardiovascular services for patients with cancer.

Importantly, integration does not require lifelong specialist management for every cancer survivor. Specialist cardio-oncology expertise is most relevant for patients with high baseline cardiovascular risk, complex cardiovascular disease that may influence cancer treatment, substantial treatment-related toxicity risk, or established cardiovascular injury; routine risk-factor management can often remain within primary care, general cardiology, oncology, or survivorship services. Established approaches such as guideline-based risk assessment, echocardiography, and conventional cardiac biomarkers should be distinguished from emerging tools such as artificial intelligence, novel biomarkers, and some digital surveillance strategies, which require prospective validation, demonstration of clinical utility, and assessment of generalizability and cost-effectiveness before routine adoption. Successful implementation should therefore be measured by cardiovascular outcomes, the ability to maintain or complete anticancer therapy, quality of life, healthcare utilization and cost-effectiveness, as well as equitable access to care.

## Conclusion

The history of cardio-oncology illustrates how medical specialties emerge—not through a single discovery but through the convergence of clinical necessity, scientific evidence, professional collaboration, and institutional commitment. Today, cardio-oncology possesses internationally recognized guidelines (5), specialized clinical programs (25), multidisciplinary care models (8), dedicated educational initiatives (9, 28), and an expanding research enterprise. These are the defining characteristics of an established specialty rather than an emerging one.

The next chapter is no longer about proving the importance of cardio-oncology. It is about ensuring that every patient with cancer, regardless of geography or healthcare system, benefits from evidence-based cardiovascular care integrated throughout the cancer care. In many respects, cardio-oncology has become the discipline uniquely positioned to protect two life expectancies simultaneously: survival from cancer and preservation of lifelong cardiovascular health. Realizing this vision will require standardized training and competency assessment, continued expansion of multidisciplinary clinical services, and risk-stratified integration of cardio-oncology expertise into routine cancer care pathways. Future recognition within the ABMS certification framework could further consolidate the specialty's professional identity, while its success should ultimately be judged by measurable cardiovascular, oncologic, patient-centered, health-system, and equity outcomes.

## Data Availability

The original contributions presented in the study are included in the article/Supplementary Material, further inquiries can be directed to the corresponding author.
